# Comparison of time-series models for monitoring temporal trends in endemic diseases sero-prevalence: lessons from porcine reproductive and respiratory syndrome in Danish swine herds

**DOI:** 10.1186/s12917-019-1981-y

**Published:** 2019-07-08

**Authors:** Ana Carolina Lopes Antunes, Dan Jensen

**Affiliations:** 1 Division for Diagnostics and Scientific Advice – Epidemiology, National Veterinary Institute/Centre for Diagnostics - Technical University of Denmark, Kongens Lyngby, Denmark; 2 Department of Veterinary and Animal Sciences, University of Copenhagen, Frederiksberg C, Denmark

**Keywords:** Surveillance, Time series, Modeling, Trends

## Abstract

**Background:**

Monitoring systems are essential to detect if the number of cases of a specific disease is rising. Data collected as part of voluntary disease monitoring programs is particularly useful to evaluate if control and eradication programs achieve the target. These data are characterized by random noise which makes harder to interpret temporal changes in the data. Monitoring trends in the data is a possible approach to overcome this issue.

The objective of this study was to assess the performance of three time-series models that allows monitoring trends in data in terms of its adaptability when used to monitor changes in disease sero-prevalence at a national scale based on data collected as part of voluntary monitoring programs. We compared two Bayesian forecasting methods and an Exponential smoothing method, specifically a Dynamic Linear Model, a Dynamic Generalized Linear Model and a Holt’s linear trend method, respectively. These three different types of time series models were applied to data on weekly sero-prevalence of Porcine Reproductive and Respiratory Syndrome (PRRS) in Danish swine herds.

**Results:**

Comparing the linear cross-dependence between the filtered values obtained from the three models and the raw data, we observed that the Holt’s linear trend method shows negative linear dependence for roughly half of the time for breeding/nucleus and multiplier herds, having values close to zero for most of the period in finisher herds.

**Conclusions:**

Bayesian forecasting methods adapt faster to changes in the data, compared to the deterministic Holt’s linear trend method. The practical implication of this greater flexibility is that the Bayesian methods will provide more reliable values of changes in the data and have potential to be implemented as part of a surveillance system in Denmark.

**Electronic supplementary material:**

The online version of this article (10.1186/s12917-019-1981-y) contains supplementary material, which is available to authorized users.

## Background

Monitoring systems are essential to detect changes in disease status in a timely and effective manner. Failure of control (and eradication) programs may have a devastating economic impact on herds with susceptible animals.

In the context of endemic diseases, slow and gradual increases in incidence and prevalence are expected to be observed due to the existence of vaccination and health management programs and to previous exposure, which can lead to natural immunity of several individuals in the population [1]. When following up on control and eradication programs, the failure to achieve a target value of disease prevalence within a certain period of time indicates that the implemented strategies should be revised. These programs are often based on laboratory diagnostic tests performed on a regular basis. As a result, the serological results generate large amounts of data characterized by random noise, due to the variation in the disease prevalence and the number of herds tested over time. The frequency of laboratory diagnostic testing also depends on the economic value of the animal and on the disease impact [2]. In these cases, the data differ from those obtained from traditional surveillance (generally focused on identifying new cases) increasing their complexity as disease burden is affected not only by new cases but also by the duration and recovery rate.

In recent years, several studies explored the performance of different temporal monitoring methods in detecting outbreaks of (re-)emerging diseases [3, 4]. However, these methods might result in false alarms when applied to laboratory diagnostic data characterized by random noise and, as a consequence, with the costs of investigation of these alarms as well as a lower trust on the monitoring system. One alternative approach could be to monitor the trend of the underlying level of the time series, which can be positive or negative depending on whether the time series exhibits an increasing or decreasing pattern [5, 6]. This is particularly useful for monitoring temporal changes in trends laboratory diagnostic results collected as part of voluntary disease monitoring programs. Based on these data, veterinarian authorities can implement control measures whenever certain thresholds related to the disease status have been exceeded. Furthermore, the efficiency of implemented control measures and eradication programs can be evaluated and redefine whenever the disease prevalence (and incidence) fails to achieve a certain level.

In a previous simulation study [6], monitoring the trend component showed great potential as a basis for monitoring temporal changes in diseases prevalence. However, those methods were not applied to real laboratory diagnostic data collected as part of voluntary disease monitoring programs, where challenges such as shifts in the collection frequency often occur.

The objective of this study was to assess the performance of three time-series models in terms of their adaptability when used to monitor changes in disease sero-prevalence at a national scale based on real world data. We compared two Bayesian forecasting methods, namely a Dynamic Linear Model (DLM) and a Dynamic Generalized Linear Model (DGLM), both with a linear trend component. The third time series model was an exponential smoothing method, specifically a Holts’ linear trend model. These three different types of time series models were applied to data on weekly sero-prevalence of Porcine Reproductive and Respiratory Syndrome (PRRS) in Danish swine herds.

## Results

### Data description

A total of 51,639 laboratory submissions from 5095 Danish swine herds sent between 2007 and 2014 were included in the analysis, corresponding to 386 Red SPF herds, 3441 Blue SPF herds and 2174 Non SPF herds. The median (Q1 — Q3) number of weekly herds tested was 59 (50–68), 53 (42–62), 23 (19–28) for Red SPF, Blue SPF and non SPF herds respectively. The median PRRS sero-prevalence from 2010 to 2014 is 0.10 (0.06–0.14) for the Red SPF, 0.32 (0.26–0.37) for the Blue SPF and 0.35 (0.27–0.43) for Non SPF herds. For the Red SPF herds with at least one positive submission throughout the study period, the median (Q1 – Q3) number of weeks between two consecutive submissions was 4 (1–5) weeks. For the Red SPF herds with all submissions negative for PRRS, the median number of weeks between two consecutive submissions was 4 (4–5) weeks. For Blue SPF herds, the corresponding values were 37 (13–52) and 51 (45–54) while for the Non SPF herds they were 32 (3–40) and 25 (2–27) respectively.

### Models initiation and parametrization

The DLM was run with deltas of 0.95, 0.97 and 0.98 for Red, Blue and Non SPF herds respectively; deltas of 0.98, 0.97 and 0.98 were used in the DGLM for the same herd types respectively. For PRRS sero-prevalence in Red SPF herds, the initial trend was − 0.0004 with an initial level of 0.129 and a beta of 0.001 and alpha of 0.08 for the Holt’s linear trend method; an initial trend of 0.0004 and initial level of 0.373 with a value of beta of 0.001 and alpha of 0.21 was defined for Blue SPF herds. For modeling PRRS sero-prevalence in Non SPF herds, the Holt’s linear trend method was defined with an initial trend of − 0.0004, an initial level of 0.404 with a beta of 0.113 and alpha of 0.002.

### Modeling performance

Results of the forecast errors obtained for the three modeling approaches are shown in Table 1. The root mean of squared errors (RMSE) of the DGLM is significantly smaller than the RMSE of the DLM for Red and Non SPF herds. The RMSE of Holt’s linear trend method is significantly larger than both Bayesian forecast methods for Red SPF herds and significantly lower for Non SPF herds. For Blue SPF herds the RMSE values only differed on the 4th decimal place.

**Table 1:** Forecast accuracy comparison of modeling weekly Porcine Reproductive and Respiratory Syndrome sero-prevalence in Danish swine herds. The out of the sample period is January 2010 to December 2014. The Red SPF herds are tested on a monthly basis for PRRS; the Blue SPF herds are tested at least once a year for PRRS; the Non SPF herds are tested with other frequencies for PRRS

Root Mean of Squared errors			
Model	Red SPF herds	Blue SPF herds	Non SPF herds
DLM (95%CI)	0.061 (0.057–0.066)	0.081 (0.079–0.083)	0.128 (0.125–0.132)
DGLM (95%CI)	0.056 (0.053–0.060)	0.081(0.079–0.083)	0.124 (0.120–0.127)
HM (95%CI)	0.219 (0.204–0.235)	0.081 (0.079–0.083)	0.096 (0.093–0.099)

*CI* Confidence Intervals

*DLM* Dynamic Linear Model

*DGLM* Dynamic Generalized Linear Model

*HM* Holt‘s linear trend method

**Fig. 1:**
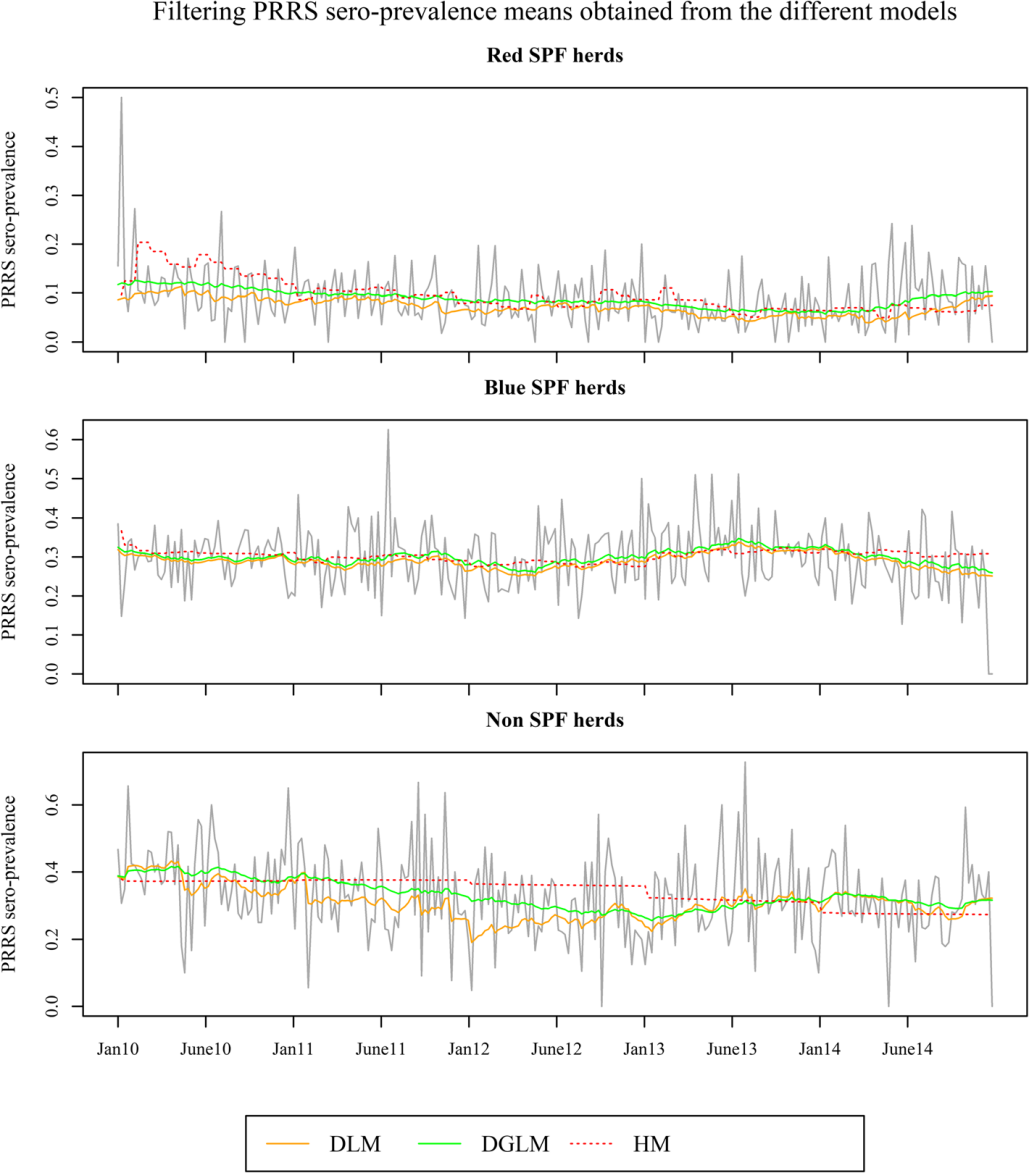
Comparison between filtered means obtained from the different models and Porcine Reproductive and Respiratory Syndrome sero-prevalence between January 2010 and December 2014. The filtering values obtained from the Dynamic Linear Model (DLM), from the Generalized Dynamic Linear Model (DGLM) and from the Holt’s linear trend method (HM) are represented for the different herd types. The Red SPF herds are tested on a monthly basis for PRRS; the Blue SPF herds are tested at least once a year for PRRS; the Non SPF herds are tested with other frequencies for PRRS

Comparing the filtered means obtained from the different models (Fig. 1), it is possible to observe that the DLM is the method with the highest flexibility to adapt to changes in PRRS sero-prevalence for the different herd types. This is most evident for the period between June 2011 and January 2013 for Non SPF herds where the filtered values of the DLM followed the decreasing shifts in PRRS sero-prevalence in contrast with the HW filtered mean, which stayed at a constant level.

### Local linear cross-dependence

**Fig. 2:**
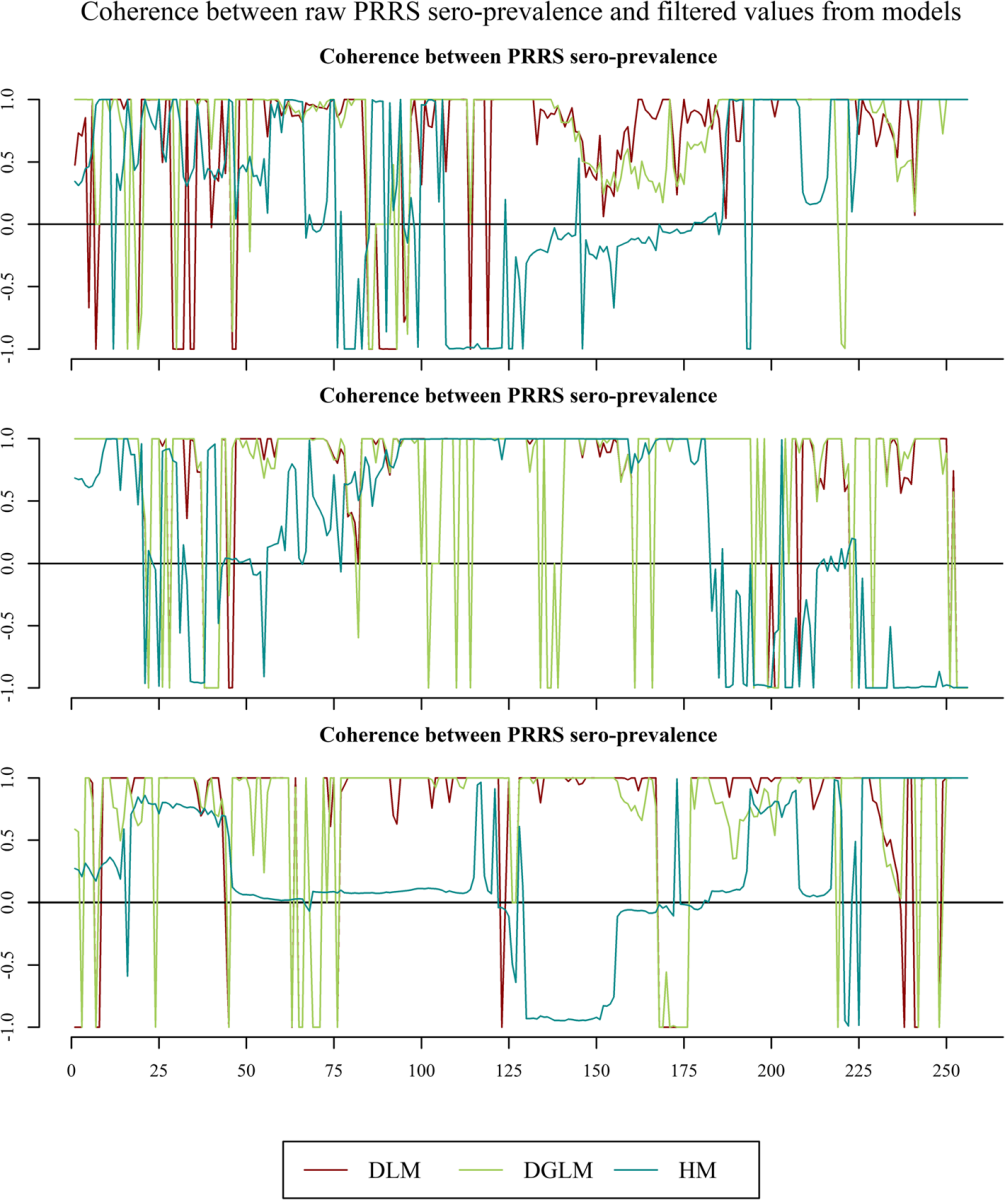
Wavelet coherence plot at scale-scale *j* = 1. The x-axis indicates specific time points (weeks) observed in the time-series since the first observation (i.e. *t* = 1 corresponds to 23rd July 2012 until 31st December 2014). The coherence obtained for the Dynamic Linear Model (DLM), from the Generalized Dynamic Linear Model (DGLM) and from the Holt’s linear trend method (HM) are represented

A Kernel smoother parameter of 4 for the DLM and DGLM and of 30 for the Holt’s linear trend method were found to minimize the generalized cross-validation gamma deviance of the multivariate Evolutionary Wavelet Spectrum analysis. Figure 2 illustrates the local linear cross-dependence between PRRS sero-prevalence in the different herd types and the filtered values obtained from the three different models for level *j* = 1, i.e. the wavelet should include 1 time unit corresponding to 1 week. The local linear cross-dependence between PRRS sero-prevalence in the different herd types and the filtered values obtained from the DLM and the DGLM show a positive linear dependence for most of the time (i.e. the filtered values obtained from these models followed the observed changes in PRRS sero-prevalence). The linear cross-dependence between PRRS sero-prevalence in the different herd types and the filtered values obtained from the Holt’s linear trend method shows a negative linear dependence for roughly half of the time for Red and Blue SPF herds, having values close to zero for most of the period in Non SPF herds. The sum of the local linear cross-dependence absolute values obtained for the whole period of study (256 weeks starting from 23rd July 2012 to 31st December 2014) are presented in Table 2.

**Table 2:** Sum of the local linear cross-dependence absolute values observed between 23rd July 2012 and 31st December 2014

Model	Red SPF herds	Blue SPF herds	Non SPF herds
DLM	223.55	234.32	242.33
DGLM	213.81	234.68	229.74
HM	159.51	191.24	113.46

*DLM* Dynamic Linear Model

*DGLM* Dynamic Generalized Linear Model

*HM* Holt‘s linear trend method

## Discussion

The performance of three time-series models for monitoring trends in PRRS sero-prevalence in different types of Danish swine herds was assessed. Bayesian forecasting methods demonstrated more flexibility to adapt to shifts in PRRS sero-prevalence time-series when compared to the Holt’s linear trend method.

The Red SPF herds are tested for PRRS on a monthly basis, while for Blue SPF herds the frequency of testing is once per year and Non SPF herds have no legal requirements for testing frequency. For Red SPF and Blue SPF herds, the laboratory diagnostic data are a good indication of the between-herd PRRS sero-prevalence at country level. For Non SPF herds, the frequency of testing depends on suspicions of outbreaks, farmer compliance, trading purposes and ongoing control and eradication programs. This resulted in time-series characterized by random noise, as a result of the variation in the disease prevalence and the number of herds tested over time, as well as larger shifts on the level, as seen in Fig. 1.

One core feature, which separates the two Bayesian methods from the Holt’s linear trend method, is that the Bayesian methods are fundamentally stochastic in their way of updating the parameter vector, while the Holt’s linear trend method is fundamentally deterministic; in the Bayesian approach, the values in the parameter vector are updated at each time step, based not only on the magnitude of the forecast errors but also the level of confidence we can have in the forecast error, expressed as the forecast variance in the Kalman filter [7]. Furthermore, the observational variance, which is used when calculating the forecast variance [7] was dependent on the number of herds observed at a given time step. In other words, the amount by which the model would error-correct was influenced by the reliability of the observed prevalence values, as well as the reliability of the model-based forecasts and forecast errors. With all of these factors, which went into the Bayesian estimation of the most likely underlying true levels of the time series, it is perhaps not surprising that both of the Bayesian methods showed greater flexibility than the deterministic method. This is particularly useful to veterinarian authorities to provide reliable information when implementing (and revising) disease control and eradication programs whenever certain thresholds related to the disease status have been exceeded. Also, in cases where a shift occurs from voluntary to mandatory surveillance programs (or vice versa), it would be expected that the variance of the prevalence would be significantly affected; for the mandatory records, you would expect that the farms being tested are much the same from one month to the next, leading to less variation. The Bayesian methods would be able to take such changes into account when estimating the true prevalence, while this would be impossible for deterministic methods such as the Holt’s linear trend method.

## Conclusions

We showed that Bayesian forecasting methods adapt faster to changes in the data, compared to the deterministic Holt’s linear trend method. The practical implication of this greater flexibility is that the Bayesian methods will provide more reliable values of changes in the prevalence and have the potential to be implemented as part of a surveillance system in Denmark. Understanding these differences in utility of various monitoring methods will allow veterinarian authorities to improve the implementation (and revisions) of disease control and eradication programs at national scales.

## Methods

### Data sources

Laboratory submission data stored in information management systems in the National Veterinary Institute - Technical University of Denmark and in the Laboratory for Swine Diseases - SEGES Pig Research Centre were used to determine the weekly PRRS sero-prevalence in Danish swine herds from January 2007 to December 2014. The laboratory diagnostic test results for PRRS from both laboratories were only available for this period of time. Collections of individual blood samples collected on the same day from different animals from a given herd are processed as individual laboratory submissions. Only submissions with at least 10 individual blood samples tested by serological tests (Blocking Enzyme-Linked Immunosorbent Assay — ELISA ([8]; IDEXX, Ludwigsburg, Germany) and/or Immunoperoxidase monolayer assay — IPMA [9]) were included in the study. Herds were classified as PRRS sero-positive when at least 2 individual blood samples in each submission tested PRRS positive. The country-level between-herd PRRS sero-prevalence was calculated weekly as the proportion of PRRS positive herds for a given time period from the total number of herds tested for PRRS on that week. This will be referred to as “PRRS sero-prevalence” throughout the manuscript.

In Denmark, the Specific Pathogen Free (SPF) system is a voluntary health program with established rules for monitoring several diseases, including PRRS [10]. The designations “Red”, “Blue” and “Green” are used within the SPF system to classify the herds according to its biosecurity status. Within the SPF herds, the “Red” herds are tested every month for PRRS and the “Blue” and “Green” herds are tested at least once a year. The laboratory submission data were merged with the SPF database and, based on the herd id numbers, the herds were classified as Blue, Red and Non SPF herds (i.e. if the herds were not part of the SPF system). Due to the low number of “Green” SPF herds in the SPF system, these herds were included in the analysis as Blue SPF herds. The individual time-series for each herd type were created and modeled separately. The seasonality test was performed based on auto correlation plots (ACF function in R) of individual time-series and by comparing the fitness of the Holt Winters model with and without seasonal components (both “additive” and “multiplicative”). No autocorrelation or seasonality was found in the time series.

The datasets for this manuscript are not publicly available because are owned by the National Veterinary Institute — Technical University of Denmark and by the Pig Research Centre–SEGES.

### Modeling

All methods described below were performed in R (version 3.5.0) [11].

#### Bayesian forecasting methods

Two Bayesian forecast models, a DLM and a DGLM, both with a linear trend, as described by [6] were used to model the different time-series. The aim of these models is to estimate the underlying true value combining the observed data (i.e. PRRS sero-prevalence) with a conditional distribution *ϴ*_*t*_ given by D_*t*_ (*ϴ*_*t*_| D_*t*_) sequentially for each time step. A linear growth component includes a time-varying slope (or local linear trend) was incorporated in the model to allow the system to adapt to a possible positive or negative growth for each *t*.

A DLM model is represented by a set of two equations, defined as the observation equation (Eq. 1) and the system equation (Eq. 2).

1 $$ {Y}_t={F}^{\hbox{'}}.\kern0.5em {\theta}_t+{v}_t,\kern0.5em {v}_t\sim N\left(0,{V}_t\right) $$

2 $$ {\theta}_t=G.\kern0.5em {\theta}_{t-1}+{w}_t,\kern0.5em {w}_t\sim N\left(0,{W}_t\right) $$

where *V*_*t*_ and *W*_*t*_ are referred to as the observational variance and system variance, respectively. The transposed design matrix (***F***^′^) had the following structure:

3 $$ \kern1.75em {\boldsymbol{F}}^{\prime }=\left[1\kern0.5em 0\right] $$

Eq. 2 describes the evolution of ***ϴ*** from time *t*-1 to *t*. The system matrix (***G***) for a local linear trend model is given as:

4 $$ \kern1em \boldsymbol{G}=\left[\begin{array}{cc}1& 1\\ {}0& 1\end{array}\right] $$

In our study, the observational variance was adjusted for the number of submissions in a given week:

5 $$ {v}_t=\frac{Y_{t-1}\ \left(\ 1-{Y}_{t-1}\right)}{n_t} $$

where *n*_*t*_ was the number of herds tested for PRRS on that week.

Unlike the DLM, the DGLM was based on a binomial distribution. The observation equation for the DGLM was defined as:

6 $$ {P}_t={F}^{\prime }.\kern0.5em {\theta}_t $$

For both models, the system variance (*w*_*t*_), which described the evolution of variance-covariance of the system for each time step, was modelled using a discount factor (δ), as previously described by [7].

The parameter vector was updated according to Bayesian principles. This was achieved with model-specific implementations of the Kalman filter, as described in detail by [7].

In order to account for value of zero in PRRS sero-prevalence, the parameter vector would be updated deterministically in accordance with eq. 2, but the stochastic, i.e. Bayesian, aspects of the update [7] would be omitted.

The DLM and DGLM were both programmed and implemented as described by [6]. The R code used for each model can be found as Additional file 1. The same implementation of the DLM and DGLM was validated on simulated data in a previous study [6]. These models have further been validated and used in previous studies [12–15].

#### Exponential smoothing method

The Holt linear trend method was also used to model the different time-series. The method is described by [16] and is an exponentially weighted moving average filter of the level and trend components of a time series. Briefly, the Holt’ linear trend method allows forecasting of data with a trend. The level (*l*) and the trend (*b*) are then updated with the following equations:

7 $$ {l}_t=\upalpha\ {Y}_t+\left(1-\upalpha \right)\left({l}_{t-1}+{b}_{t-1}\right) $$

8 $$ {b}_t=\upbeta\ \left({l}_t-{l}_{t-1}\right)+\left(1-\upbeta \right){b}_{t-1} $$

and the k-step ahead forecast is then:

9 $$ {\hat{Y}}_{t+h\mid \mathrm{t}}={l}_t+{b}_tk $$

where *l*_*t*_ denotes an estimate of the level of the series at time t, *b*_*t*_ denotes an estimate of the trend (or growth) of the series at time t, α is the smoothing parameter for the level (0 < α < 1), β is the smoothing parameter for the trend (0 <  β < 1).

The Holt’s linear trend method was used based on the ‘HoltWinters’ function in R.

#### Models initialization and parameterization

Reference analysis was used to estimate the initial parameters *D*_0_~[*m*_0_, *C*_0_] for the DLM and the DGLM as described by [7]. The DLM and the DGLM models were run on the data between 2007 and 2009 with different values for δ ranging from 0.1 up to 1 by increments of 0.01. The discount factor which minimized the sum of the squared forecast errors was chosen for the analysis. For the DLM and the DGLM, the forecast errors *e*_*t*_, standardized with respect to their variance Q_t_, such ${u}_t={e}_t/\sqrt{{\mathrm{Q}}_{\mathrm{t}}}$

In order to estimate the initial trend to initiate the Holt’s linear trend method, a subset of the sero-prevalence for each herd type in 2007 was used. A linear model was used to estimate this initial declining trend where the week of each year was used as predicted variables for PRRSV sero-prevalence. The initial level was estimated as the average sero-prevalence in 2007 for each herd type. -.

The different combinations of the smoothing parameters α and β ranging from 0 up to 1 with increments of 0.00001 where tested in the different subsets with data between 2007 and 2009. The combination of α and β which minimized the sum of the squared forecast errors for the Holt’s linear trend method was used.

#### Modeling performance

The model fitting was evaluated using the following quantitative measures [7]:

10 $$ Root\ Mean\ Squared\ Error\ (RMSE)=\kern0.5em \sqrt{\kern0.5em \frac{\sum_{t=1}^i{e_t}^2\ }{N}} $$

, where *e*_*t*_ is forecast errors and *N* is the total number of observations in data from 1st January 2010 to 31st December 2014.

Additionally, the 95% confidence intervals (CI) for RMSE were calculated, assuming that the prediction errors were normally distributed around a mean of 0 with a constant standard deviation for all forecast errors. Thus, the χ^2^ distribution could be used to estimate the CI as:

11 $$ 95\%{CI}_{RMSE}=\left[\sqrt{\frac{n}{\chi_{1-\frac{5}{2},n}^1}}.\kern0.5em RMSE,\sqrt{\frac{n}{\chi_{\frac{5}{2},n}^1}}.\kern0.5em RMSE\ \right] $$

, where *n* is the number of degrees of freedom, equivalent to the number of herds included in the study for each herd-type.

### Linear cross-dependence

We used the method described by [17] as it provides a measure of the presence (or lack) of local linear cross-dependence between PRRS sero-prevalence and the filtered values obtained from the three different models for each time step *t* and level *j*; the level expresses how many time units the wavelets should be “stretched over” and it can be interpreted as the time-series lag. The functions ‘mvEWS’ and ‘coherence’ from the R package ‘mvLSW’ (version 1.2.1) [18] were used to estimate and quantify the dynamic linear cross-dependence between PRRS sero-prevalence and filtered means obtained from the three models with a default wavelet and values ranging from 1 to 30 with increments of 1 were used as Kernel smoothing parameters. The scale of *j* = 1, which corresponds to 1 week lag, was used to evaluate the cross-dependence.

This method requires time-series with lengths of 2^*J*^ where *J* is a positive integer. Data with the latest 256 (2^8^) observations (i.e. data from 1st February 2010 to 31st December 2014) were used.

## Additional file

Additional file 1: R code used for a Dynamic Linear Model (DLM) and a Dynamic Generalized Linear Model (DGLM), both with a linear trend component. (TXT 5 kb)

## Data Availability

The data that support the findings of this study are available from the National Veterinary Institute - Technical University of Denmark and in the Laboratory for Swine Diseases - SEGES Pig Research Centre but restrictions apply to the availability of these data and so are not publicly available. Data are however available from the authors upon reasonable request and with permission of the National Veterinary Institute - Technical University of Denmark and in the Laboratory for Swine Diseases - SEGES Pig Research Centre.

## Abbreviations

CI: Confidence Intervals
DGLM: Dynamic Generalized Linear Model
DLM: Dynamic Linear Model
HM: Holt’s linear trend method
PRRS: Porcine Reproductive and Respiratory Syndrome
